# Physical function and perceived pain following inpatient intensive interdisciplinary pain treatment for children and adolescents

**DOI:** 10.1002/pmrj.13325

**Published:** 2025-01-10

**Authors:** Mayank Seth, Kate Vieni, Kathryn Hottinger, Katherine Bentley

**Affiliations:** ^1^ Research Department Children's Specialized Hospital Mountainside New Jersey USA; ^2^ Department of Physical Medicine and Rehabilitation Rutgers New Jersey Medical School Newark New Jersey USA; ^3^ Physical Therapy Children's Specialized Hospital New Brunswick New Jersey USA; ^4^ Psychology Children's Specialized Hospital New Brunswick New Jersey USA; ^5^ Physiatry Children's Specialized Hospital New Brunswick New Jersey USA

## Abstract

**Background:**

Chronic pain among children and adolescents negatively impacts overall functioning and quality of life. Although Intensive Interdisciplinary Pain Treatment (IIPT) programs aim to reduce functional impairment and perceived pain, overall evidence is limited and restricted by small sample sizes and limited diversity in pain diagnoses.

**Objective:**

To determine whether children and adolescents with chronic pain participating in an inpatient IIPT program experience improvements in their physical function and perceived pain.

**Design:**

Cross‐sectional, secondary analysis.

**Setting:**

Inpatient acute rehabilitation.

**Participants:**

Children and adolescents with chronic pain (*n* = 258; females/males = 204/54; age = 16.5 ± 2.6) admitted to a 4‐week inpatient IIPT program from November 2011 to January 2023.

**Intervention:**

Participants attended individual and group sessions involving physical therapy, occupational therapy, aquatic therapy, cognitive behavioral therapy, school‐related tasks, and meditation. The sessions focused on improving strength, endurance, and function, while helping participants modify physical sensations, catastrophic thinking, and maladaptive behaviors.

**Main Outcome Measures:**

Collected at admission and discharge: pain intensity (Numerical Pain Rating Scale; scale: 0–10), lower extremity function (Lower Extremity Functional Scale [LEFS]; scale: 0–80), upper extremity function (Upper Extremity Functional Index [UEFI]; scale: 0–80), motor proficiency (Bruininks–Oseretsky Test of Motor Proficiency, Second Edition short form [BOT‐2 SF]; scale: 0–70), and occupational performance and satisfaction (Canadian Occupational Performance Measure [COPM]; scale: 0–10 for both).

**Results:**

Overall, participants reported significant improvements (*p* < .05) in median LEFS (median change [MC] = +30.5; 25th, 75th percentile range [PR] = 19, 42), UEFI (MC = +21; PR = 12.8, 31), BOT‐2 SF (MC = +9; PR = 5, 15), COPM performance (MC = +4; PR = 2.8, 5.4), and COPM satisfaction (MC = +5.6; PR = 3.8, 7.2). Moreover, participants reported significant reduction (*p* < .05) in median pain intensity (MC = −3; PR = 1, 5). For a majority of participants, MC surpassed previously reported minimally clinical important difference thresholds.

**Conclusions:**

Findings highlight the relevance of inpatient IIPT programs in enhancing physical function and reducing perceived pain in children and adolescents with chronic pain.

## INTRODUCTION

Chronic pain, defined as pain lasting beyond the anticipated healing period (>3 months), is a debilitating condition affecting a vast population across their life span.^1^, ^2^ According to previous estimates up to 35% of children may experience chronic pain, with 17% to 35% of adults with chronic pain reporting first experiencing pain during their childhood.^2^ Moreover, 5% to 8% of children may experience severe disability as a result of chronic pain.^3^ Children with chronic pain often experience poor outcomes affecting various aspects of their daily life, including personal, academic, social, and recreational.^1^ For example, functional impairment limits their ability to perform daily tasks independently and attend school.^1^, ^4^ Consequently, children with chronic pain are at an elevated risk of experiencing anxiety, depression, and an overall diminished quality of life.^5^, ^6^

Chronic pain and subsequent impairments result from an interaction of several factors, including neurosensory, physical, behavioral, cognitive, and sociocultural.^1^, ^7^ The individual contribution of each factor toward patient outcomes, however, remains unclear.^1^ Unlike acute pain, pharmacological interventions have not been shown to be as effective in treating chronic pain and have potential adverse side effects.^8^, ^9^ Therefore, chronic pain treatment and management is best approached with a biopsychosocial model of care (eg, intensive interdisciplinary pain treatment [IIPT]) where the focus is not on reducing pain intensity via pharmacological interventions but rather on helping to improve function and quality of life.^1^, ^7^, ^10^ Given the role function plays in an individual's ability to perform various daily activities, participate in social roles, and overall health and well‐being, regaining lost function is an important part of patient rehabilitation.^11^, ^12^ Hence, an important emphasis of IIPT is on improving function that may be impaired either from the pain itself or fear‐related avoidance.^4^, ^13^ This is achieved via muscle strengthening and endurance exercises, progressively reengaging in previously avoided activities, and helping patients develop better coping strategies.^14^, ^15^

The impact of IIPT on physical function is well evidenced in the adult population.^16^, ^17^ Although literature suggests improvements in physical function in children and adolescents following IIPT,^18^, ^19^, ^20^ overall evidence is constrained by several factors. First, there is a scarcity of studies that delve into comprehensive assessments of function, incorporating self‐reported and performance‐based function, along with goal‐oriented assessments. Second, much of the literature is focused on specific chronic pain diagnoses like complex regional pain syndrome or juvenile fibromyalgia, limiting generalizability to the broader and more heterogeneous chronic pain population typically encountered in clinical practices and IIPT programs. Lastly, research in this area is limited by relatively small sample sizes, making it challenging to draw robust conclusions. Considering the substantial costs associated with each episode of IIPT (ie, >$30,000 per episode, which typically lasts for a few weeks),^16^ further research is imperative to assess its efficacy in improving physical function among children with chronic pain. Hence, the purpose of this study is to determine whether children and adolescents participating in an inpatient IIPT program experience changes in their lower and upper extremity function, motor proficiency, occupational performance, and satisfaction, as well as perceived pain. We hypothesized significant improvements in all these outcomes following discharge from IIPT program. Our findings may inform future clinical trials and longitudinal studies aiming to mitigate outcomes in children with chronic pain.

## METHODS

Data for this secondary analysis were extracted from a preexisting dataset obtained from an inpatient IIPT program at Children's Specialized Hospital, New Brunswick, New Jersey. For the IIPT program, patients were admitted if they were between the ages of 9 a 22 years, had a chronic pain diagnosis (ie, pain lasting ≥3 months), and had previously received outpatient treatment for chronic pain that was unsuccessful in alleviating symptoms. Individuals were not considered for admission if they had suicidal ideation or attempted suicides in the past 6 months, and had a cognitive, neurological, or musculoskeletal condition limiting their participation in the program. All participants who received clinical care in the program from November 2011 to January 2023 were considered for inclusion in this secondary analysis. Potential participants were excluded only if they had missing data. This secondary analysis was approved as an exempt study by the Western‐Copernicus Group Institutional Review Board (Project #: 45330799; Approval Date: 02/09/2023).

### IIPT protocol

Participants completed an IIPT program at Children's Specialized Hospital, with an average length of stay of 4 weeks. IIPT programs are recognized for their comprehensive, multimodal approach to chronic pain, focusing on physical, cognitive, emotional, and behavioral aspects of pain management.^1^, ^7^, ^10^ These programs aim to enhance strength, endurance, and function while promoting psychological resilience, in line with well‐established models of care used across the United States and internationally.^21^, ^22^, ^23^, ^24^

At Children's Specialized Hospital, patients engaged in 7 to 8 hours of daily therapy (5 days a week), consisting of both individual and group sessions. The treatment program included physical therapy, occupational therapy, aquatic therapy, cognitive behavioral therapy, school‐related tasks, and meditation. The following is a breakdown of an example daily schedule:

- Aquatic therapy: Each day began with a 1‐hour group aquatic therapy session, focusing on functional strength, balance, and weight‐bearing activities for the lower extremities.
- Meditation: This was followed by a 30‐minute group meditation session designed to promote mindfulness and relaxation. Each session was led by professionals from psychology and various therapies, offering different meditation types to promote relaxation and well‐being.
- Occupational therapy (OT): Individualized OT sessions concentrated on functional improvements and pain management. OT addressed desensitization techniques for extremities affected by allodynia, structured daily tasks to improve task persistence, movement quality, and speed, and cognitive‐based activities to boost concentration and focus. Patients received 2–3 hours of OT daily.
- Cognitive behavioral therapy (CBT): Twice a week, after OT, patients participated in 1‐hour CBT sessions with a psychologist. These sessions targeted coping strategies to modify catastrophic thinking and maladaptive behaviors.
- Physical therapy (PT): Individualized PT sessions targeted endurance, task persistence, movement quality, and strength. Strengthening exercises utilized bands, weights, and body weight, and functional strengthening focused on gross motor tasks. Endurance training incorporated equipment like treadmills, ellipticals, and bikes. Additionally, patients worked on positional tolerance (sitting, standing) on various surfaces to enhance balance and weight‐bearing on the affected side. Patients received 2–3 hours of PT daily.
- School‐related activities: Twice a week, patients participated in group PT and OT sessions focused on exercise and school‐related tasks such as reading, writing, and typing, followed by a group study session. On other days, patients were given individual study breaks to continue working on schoolwork.
- Education sessions: Participants attended comprehensive pain education sessions, providing them with a deeper understanding of pain mechanisms, management strategies, and coping techniques tailored to their condition.
- Family meetings and training: Weekly family meetings were held with the interdisciplinary team and parents to discuss the patient's progress, address concerns, and adjust treatment goals. Additionally, parents participated in family education sessions designed to equip them with skills to support their child's pain management and rehabilitation at home.

This comprehensive interdisciplinary program emphasizes an individualized approach that addresses both the physical and psychological components of chronic pain, aiming to achieve long‐term functional improvements for children and adolescents.

### Outcomes

A member of the clinical team obtained information on demographics (ie, age, gender, race), pain diagnosis, comorbidities (ie, history of injury, history of psychological diagnosis), pain medication at admission and discharge, and use of assistive technology at admission and discharge. As part of a standardized clinical evaluation, participants completed patient‐reported outcomes.

#### Pain intensity

The Numerical Pain Rating Scale (NPRS) is a valid measure of pain intensity among children with chronic pain.^25^ The NPRS is an 11‐point scale (0–10) where 0 indicates no pain and 10 indicates worst imaginable pain. Each patient rated their pain intensity at the time of admittance to the IIPT, discharge, and 12‐month follow‐up.

#### Physical function

The following functional outcomes were completed by participants at admittance and discharge from the IIPT program.

The Lower‐Extremity Functional scale (LEFS) has been demonstrated in previous research to be a valid and reliable measure (test–retest intraclass correlation coefficient [ICC]_2,1_ = 0.86) for assessing an individual's lower extremity function in various activities.^26^ The LEFS consists of 20 items, each scored on a 5‐point scale (0–4), with 0 indicating “extreme difficulty or unable to perform activity” and 4 indicating “no difficulty.” Scores on all items are summed for a maximum possible score of 80. Higher scores are indicative of greater lower extremity function.^27^

The Upper Extremity Functional Index (UEFI) has been demonstrated in previous research to be a valid and reliable measure (test–retest ICC_2,1_ = 0.94) for assessing an individual's upper extremity function on various activities.^28^ The UEFI consists of 20 items each scored on a 5‐point scale (0–4), with 0 indicating “extreme difficulty or unable to perform activity” and 4 indicating “no difficulty.”^28^ Scores on all items are summed for a maximum possible score of 80.^28^ Higher scores are indicative of greater upper extremity functional status.^28^

The Bruininks–Oseretsky Test of Motor Proficiency, Second Edition short form (BOT‐2 SF) has been demonstrated in previous research to be a valid and reliable measure (test–retest ICC_2,k_ = 0.97) of fine and gross motor proficiency.^29^, ^30^ The test consists of 14 items assessing performance on eight subdomains: fine motor precision (drawing lines through crooked paths, folding paper), fine motor integration (copying a square, copying a star), manual dexterity (transferring pennies), bilateral coordination (jumping in place [same side synchronized], tapping feet and fingers [same side synchronized]), balance (walking forward on a line, standing on one leg on a balance beam [eyes open]), upper limb coordination (dropping and catching a ball with both hands, dribbling ball with alternating hands), strength (knee push‐ups, sit‐ups), and speed and agility (jumping on one leg).^31^ Raw score on each item is converted into a point score, which is further converted to a standard score by comparing with normative data. Higher scores are indicative of greater motor proficiency.^32^

The Canadian Occupational Performance Measure (COPM) has been demonstrated in previous research to be a valid client‐focused measure to assess change in an individual's perceived occupational performance.^27^, ^33^ The COPM consists of a semistructured interview process where a trained occupational therapist interviews the patient on activities that are important to them.^33^ After identifying five of the most important activities, patients are asked to score them on a 10‐point scale (1 – 10), where 1 indicates they are “not at all able” to perform the activity, and 10 indicates they are “able to perform the activity extremely well”.^33^ The score on each activity is averaged to get the patient's COPM Performance rating.^33^ Additionally, patients are also asked to provide a satisfaction rating for each of the five important activities on a 10‐point scale (1–10), where 1 indicates they are “not at all satisfied” with that activity, and 10 indicates they are “extremely satisfied”.^33^ Satisfaction scores on all five activities are averaged to get the COPM Satisfaction rating.^33^ An increase in performance and satisfaction rating over time is indicative of an improvement in the patient's perception of their occupational performance.^27^, ^33^

### Statistical analysis

All statistical analyses were performed using SPSS version 29 (IBM Corp., Armonk, NY USA). Descriptive statistics (mean, SD, frequency distribution) were used to summarize participant characteristics. The normality of continuous variables was assessed using the Shapiro–Wilk test, and outliers were identified by examining boxplots. Homogeneity of variance was evaluated with Levene's test to ensure that assumptions for parametric tests were met.

Given that the data violated the assumptions of normality, the Wilcoxon signed‐rank test, a nonparametric alternative to the paired *t*‐test, was used to analyze changes in pain intensity and functional outcomes from admission to discharge. This test was chosen as it compares paired samples without assuming normal distribution. Results are presented as median values with percentile ranges to reflect the nonparametric nature of the data. Effect size was calculated using Cohen's *r*, where *r = Z/√n*, with *Z* representing the test statistic from the Wilcoxon signed‐rank test and *n* being the sample size. Cohen's *r* was interpreted following established guidelines: values of 0.1, 0.3, and 0.5 were considered small, medium, and large effects, respectively.^34^ For all planned analysis significance was accepted at *α* ≤.050.

## RESULTS

### Participants

Overall, 371 children and adolescents with chronic pain participated in the IIPT from November 2011 to January 2023. Of these, 113, were missing data on their pain rating at either admission or discharge and were excluded from the analysis, leaving 258 participants in the final analysis. Given the secondary nature of the study, a formal sample size estimation was not conducted; however, the sample size is comparable to prior literature on this topic.^18^, ^26^, ^27^ The demographic characteristics of the participants are presented in Table 1. Consistent with prior literature, the majority (ie, 79.1%) of participants were females, and the most common pain diagnosis was amplified musculoskeletal pain syndrome. Whereas most participants (ie, 58.0%) did not have any history of injury, the majority (ie, 67.8%) reported having a psychological diagnosis such as anxiety or depression. At admission, 45.7% and 33.5% participants reported taking pain medication and using assistive devices, respectively. At discharge, 8.5% and 2.8% participants reported taking pain medication and using assistive devices, respectively.

**TABLE 1:** Participant demographics (*n* = 258).

Measures	Mean (± SD)
Age (years)	16.5 (2.6)
*Range*	9–22
Length of stay (weeks)	4 (1.4)
*Range*	2–13

	Frequency (%)
Gender	
Male	54 (20.9%)
Female	204 (79.1%)
Race	
White	207 (84.5%)
Black or African American	10 (4.1%)
Asian	6 (2.4%)
American Indian or Alaskan Native	1 (0.4%)
Other or multiple races	21 (8.6%)
*Total valid cases*	*245*
Pain diagnosis	
CRPS	70 (27.2%)
Fibromyalgia	3 (1.2%)
AMPS	118 (45.7%)
Chronic headache	2 (0.8%)
Ligament laxity	1 (0.4%)
Functional abdominal syndrome	1 (0.4%)
Other	1 (0.4%)
Two pain diagnosis	35 (13.6%)
Three or more pain diagnosis	27 (10.5%)
History of injury	
No	149 (58.0%)
Yes	108 (42.0%)
*Total valid cases*	*258*
Psychological diagnosis^a^	
No	83 (32.2%)
Yes	175 (67.8%)
Pain medication	
Admission	
No	140 (54.3%)
Yes	118 (45.7%)
*Total valid cases*	258
Discharge	
No	225 (91.5%)
Yes	21 (8.5%)
*Total valid cases*	246
Assistive device use	
Admission	
No	171 (66.5%)
Yes	86 (33.5%)
*Total valid cases*	257
Discharge	
No	244 (97.2%)
Yes	7 (2.8%)
*Total valid cases*	251

*Note*: Total valid cases represent cases for which data was available on the measure.

Abbreviations: AMPS, amplified musculoskeletal pain syndrome; CRPS, complex regional pain syndrome.

^a^ Includes diagnosis of anxiety, depression, conversion disorder, attention‐deficit hyperactivity disorder, obsessive compulsive disorder, bipolar disorder.

Participant pain and functional outcomes are presented in Table 2. Following participation in the IIPT program, participants reported significant improvements in their lower and upper extremity function, per the LEFS (*p*  <.001; effect size, *r* = − 0.85) and UEFI (*p*  <.001; effect size, *r* = − 0.85), respectively, and performance‐based motor proficiency, per the BOT‐2 SF (*p*  <.001; effect size, *r* = −0.79). Participants further reported significantly greater occupational performance and satisfaction, per the COPM (Performance [*p*  <.001; effect size, *r* = − 0.87]; Satisfaction [*p*  <.001; effect size, *r* = − 0.83]). In addition to functional outcomes, participants reported significant reductions in their perceived pain intensity, per the NPRS (*p*  <.001; effect size, *r* = − 0.73).

**TABLE 2:** Participant (*n* = 258) pain and functional outcomes at admission and discharge.

Measure	Assessment time‐point		Median change^a^	Sig.	ES
	Admission	Discharge			
NPRS (0–10)	7 (6–8)	4.0 (2–6)	−3 (1–5)	<.001	−0.73
LEFS (0–80)	35.5 (24.0–47.0)	70.0 (60.0–76.0)	+30.5 (19.0–42.0)	<.001	−0.85
Total valid cases	226	228	224		
UEFI (0–80)	49.0 (39.0–59.3)	74.0 (67.0–77.0)	+21.0 (12.8–31.0)	<.001	−0.85
Total valid cases	158	158	154		
BOT‐2 SF (0–70)	39.0 (35.0–46.0)	51.0 (43.3–56.0)	+9 (5.0–15.0)	<.001	−0.79
Total valid cases	236	228	227		
COPM performance (1–10)	3.6 (2.8–4.6)	8.0 (7.2–8.9)	+4.0 (2.8–5.4)	<.001	−0.87
Total valid cases	197	193	193		
COPM satisfaction (1–10)	2.2 (1.4–3.2)	8.4 (6.6–9.4)	+5.6 (3.8–7.2)	<.001	−0.86
Total valid cases	196	191	191		

*Note*: All data presented as median (25th, 75th percentile). Total valid cases represent cases for which data was available on the measure. Significance accepted at ∝ ≤ 0.05. Effect size calculated as *r* = *Z*/√*n*.

Abbreviations: BOT‐2, Bruininks‐Oseretsky Test of Motor Proficiency, Second Edition, Short Form; COPM, Canadian Occupational Performance Measure; ES, effect size; LEFS, Lower‐Extremity Functional Scale; NPRS, Numerical Pain Rating Scale; Sig, significance; UEFI, Upper Extremity Functional Index.

^a^ Negative value indicates a decrease in the outcome from admission to discharge; Positive values indicate an increase in the outcome from admission to discharge.

## DISCUSSION

Children experiencing chronic pain may demonstrate reduced or impaired physical function. Participation in an inpatient IIPT program is aimed at enhancing patient function, thereby reducing perceived pain intensity.^4^ According to our findings, children with chronic pain participating in the inpatient IIPT at Children's Specialized Hospital demonstrated significant improvements in their self‐reported physical function (per the LEFS and UEFI), motor proficiency (per the BOT‐2 SF), and occupational performance and satisfaction (per the COPM). Moreover, significant improvements were also noted in self‐perceived pain intensity (per the NPRS). More important, changes observed between initial admission and the program's completion surpassed previously reported thresholds for clinically meaningful improvements in function and pain, suggesting these improvements may have practical implications for real‐world situations. Limited prior evidence suggests that for the majority of participants attending IIPT, improvements in pain‐related outcomes, psychological well‐being, and school performance are sustained at long‐term follow‐ups, such as at 1 year and beyond 4 years.^21^, ^22^, ^23^, ^24^ These findings support the efficacy of IIPT in achieving long‐term benefits. However, the evidence remains limited regarding the key factors that influence these outcomes,^23^ highlighting the need for further research to better understand the mechanisms driving sustained improvements.

Lower extremity function plays a significant role in the ability to perform several daily activities, such as tying shoelaces, walking a few blocks, and participating in recreational activities.^26^ Additionally, lower extremity function (ie, muscle strength) has been associated with balance and fall risk.^35^, ^36^ According to our study findings, children and adolescents with chronic pain reported significant improvements in their lower extremity function following participation in the IIPT. Moreover, for a majority of participants, 91.5%, the median change from admission to discharge, surpassed previously reported meaningful clinically important difference (MCID) threshold of 9 points for LEFS in adults with lower‐extremity musculoskeletal conditions.^26^ Our findings are consistent with prior literature. For example, Kempert et al. and Mirek et al., in a sample of children with various chronic pain diagnosis (*n* = 109) and chronic musculoskeletal pain (*n* = 173), respectively, observed significant improvements in LEFS scores following IIPT.^18^, ^27^

Similarly, following IIPT, participants reported significant improvements in upper extremity function, with the majority (81.8%) reporting improvements beyond previously reported MCID threshold of 8.4 points for UEFI in adults with upper extremity dysfunction.^28^ Our findings align with prior research; Kempert et al. observed significant improvements in upper extremity function among children with chronic pain following their discharge from an IIPT program.^18^ Although the prevalence of upper extremity functional impairment is lower when compared to lower extremity impairments, its significance may not be underestimated. Upper extremity function plays a pivotal role in maintaining independence in daily activities such as self‐care, household chores, schoolwork, and participation in recreational activities.^37^ Additionally, it can affect the use of assistive devices. Poor upper extremity function has also been linked to negative psychological outcomes, including anxiety and depression.^38^

Motor proficiency during childhood lays the foundation of an active and healthy lifestyle, influencing physical, emotional, and social well‐being.^39^ Children with poor motor proficiency are less likely to participate in physical activity, are at an increased risk of obesity, and have hindered cognitive development and compromised academic achievement.^39^, ^40^ Limited evidence suggests pain interferes in the maintenance as well as acquisition of motor proficiency,^41^ thereby contributing to poor outcomes. According to our study findings, children with chronic pain demonstrated significant improvements in motor proficiency upon completion of the IIPT program. Moreover, 61.2% of participants reported improvements surpassing the previously reported MCID threshold of 6.53 points for the BOT‐2 SF in children with intellectual disabilities.^42^ These findings align with prior research by Sherry et al., who observed similar improvements in motor proficiency among children with juvenile fibromyalgia (*n* = 64) following intensive inpatient physical and psychosocial therapy.^20^

Collaborative or personalized goal setting, which empowers individuals to establish meaningful goals related to their specific tasks and activities, is an important aspect of patient rehabilitation.^43^ Patients experiencing chronic pain often adopt acute pain strategies such as reducing function and restricting activities, with a primary goal of pain reduction. Considering this, task‐oriented goal setting holds particular significance within IIPT, as it effectively redirects the emphasis toward achieving functional recovery.^4^ Following IIPT, participants reported significant improvements in their self‐perceived performance and satisfaction with various self‐identified occupational tasks (as assessed by the COPM). These findings suggest that, following IIPT, children may experience increased confidence in their ability to successfully undertake daily self‐care tasks, enhance productivity, and actively engage in leisure activities. However, it remains to be determined whether these improvements can be sustained in the long term.

In addition to improvements in physical function, participants attending the IIPT experienced significant reductions in self‐perceived pain intensity from admission to discharge. Moreover, when determining clinically significant reductions in pain intensity,^44^ 69.8% participants experienced at least minimal reduction (or 20%), 60.8% experienced at least meaningful reduction (or 30%), and 45% experienced at least substantial reduction (or 50%) in pain. Moreover, at discharge from the program only 8.5% participants were using pain medication, as compared to 45.7% at admission. These findings hold particular importance given the primary focus of IIPT programs is not pain reduction but rather enhancement of overall functionality.^4^ Our findings are consistent with prior literature, where participation in a multi or interdisciplinary pain management program led to significantly lower rates of perceived pain among children and adolescents with chronic pain.^18^, ^20^, ^27^ Our study findings build upon prior literature by encompassing a larger and more heterogenous sample of children and adolescents with chronic pain. Collectively, these findings underscore the significant impact of IIPT in improving function and indirectly managing chronic pain.

### Study limitations

Our study strengths include robust findings across a relatively large and heterogeneous sample of children with chronic pain diagnoses. Our findings, however, are constrained by the absence of a control group and the lack of randomization, which are essential elements for establishing the intervention's effectiveness. Given the secondary nature of the data, our findings are further limited by the lack of detailed information on prior treatments, their frequency, and the time elapsed since the last intervention. Additionally, the absence of follow‐up or long‐term data on physical function limits our ability to assess sustained outcomes. To gain a more comprehensive understanding of trends and the sustainability of functional improvements, future research should consider longitudinal data collection both during and after IIPT interventions. We further acknowledge the absence of detailed information of pain medication types (ie, opioid or nonopioid) and psychosocial measures, such as assessments of anxiety or depression. These measures could provide valuable insights into patient treatment as well as overall well‐being and should be considered in future research to offer a more comprehensive perspective. Additionally, we also acknowledge the lack of outcomes specifically addressing certain pain diagnoses, such as chronic headaches, functional abdominal syndrome, and “other pain diagnosis.” Although these cases represented only 2% of our sample, future research may consider more tailored outcomes for these conditions. Lastly, it is worth noting that, in a clinical dataset, we cannot ascertain the specific reasons for missing data, resulting in a reduction in our sample size to those with available data on most variables.

## CONCLUSIONS

Following IIPT, children and adolescents with chronic pain reported significant improvements in their self‐reported upper and lower extremity function, motor proficiency, and occupational performance and satisfaction. Findings underscore the relevance of IIPT in enhancing physical function, which may be explored in future longitudinal investigations. Additionally, findings suggest that IIPT may also contribute to a reduction in perceived pain. Given IIPT's primary focus on function enhancement, a potential relationship between improved function and reduced pain warrants further investigation in future studies.

## FUNDING INFORMATION

This research did not receive any specific grant from funding agencies in the public, commercial, or not‐for‐profit sectors.

## DISCLOSURES

The authors have no conflicts of interest to report. The material within has been presented at the 2023 Annual meeting of the American Congress of Rehabilitation Medicine, October 30–November 2, 2023, in Atlanta, GA, but has not been previously published.

This journal‐based CME activity is designated for 1.0 *AMA PRA Category 1 Credit* ^TM^. Effective January 2024, learners are no longer required to correctly answer a multiple‐choice question to receive CME credit. Completion of an evaluation is required, which can be accessed using this link, https://onlinelearning.aapmr.org/. This activity is FREE to AAPM&R members and available to nonmembers for a nominal fee. CME is available for 3 years after publication date. For assistance with claiming CME for this activity, please contact (847) 737–6000. All financial disclosures and CME information related to this article can be found on the Online Learning Portal (https://onlinelearning.aapmr.org/) prior to accessing the activity.
